# An update on the role of complement in hepatocellular carcinoma

**DOI:** 10.3389/fimmu.2022.1007382

**Published:** 2022-10-19

**Authors:** Zhijie Xiao, Charlie Lot Sum Yeung, Judy Wai Ping Yam, Xiaowen Mao

**Affiliations:** ^1^ Scientific Research Centre, The Seventh Affiliated Hospital, Sun Yat-sen University, Shenzhen, China; ^2^ Department of Pathology, School of Clinical Medicine, Faculty of Medicine, the University of Hong Kong, Hong Kong, Hong Kong SAR, China

**Keywords:** complement, hepatocellular carcinoma, tumourigenesis, metastasis, tumour microenvironment, stemness

## Abstract

As a main producer of complement, the environment in the liver is greatly affected by the complement system. Although the complement system is considered to have the ability of nonself discrimination, remarkable studies have revealed the tight association between improper complement activation in tumour initiation and progression. As complement activation predominantly occurs within the liver, the protumourigenic role of the complement system may contribute to the development of hepatocellular carcinoma (HCC). Improvement in the understanding of the molecular targets involved in complement-mediated tumour development, metastasis, and tumour-promoting inflammation in HCC would certainly aid in the development of better treatments. This minireview is focused on recent findings of the protumourigenic role of the complement system in HCC.

## Introduction

Liver cancer is a global health burden, and hepatocellular carcinoma (HCC), the most common type of liver cancer, is estimated to affect over one million people annually by 2025 (1). Omics-based technologies started the new chapter of the surveillance and treatment of HCC. The combined utilisation of proteomic- and genomic-based biomarkers contributed to the early detection and diagnosis of HCC and provided more options for the better management of HCC patients. Novel therapeutic opportunities have been revealed recently in successful clinical trials using immune checkpoint blockers, indicating that the immune system could be the most promising target to achieve a cure for cancer. However, interpatient variability is the major challenge for immunotherapy in HCC. A thorough understanding of the immune landscape will facilitate the identification of treatment targets and the design of therapeutic strategies.

## Inflammation in carcinogenesis

It is well known that the body utilises the inflammatory system to exclude nonself or dead cells; however, cancer cells have been found to hijack the inflammatory system as a defence mechanism. In the majority of cancer types, cancerous cells become exclusively nonimmunogenic to avoid immunosurveillance. Although avoidance of immune destruction was not included in the six core hallmarks of cancer (2), it has been proven to be correlated with the development of carcinogenesis by numerous studies and should be considered a core hallmark of cancer. In addition, inflammation is involved in every critical step of tumourigenesis (3) and fosters other hallmarks of cancer, such as survival, proliferation and metastasis. Most recently, Douglas Hanahan stated that to better address the complexities of the pathogenesis of cancer, two additional enabling characteristics should be included in the hallmark system, and one of them is tumour-promoting inflammation (4, 5). As an important component of tumour-promoting inflammation, the complement system is involved in the formation of tumours not only by participating in the inflammatory response during tumourigenesis and metastasis but also by actively regulating the adaptive immune response and suppressing the function of T cells (6, 7).

## Liver cancer and the complement system

The complement system is a network of soluble serum proteins, membrane-bound receptors and regulatory proteins that interacts with both the innate and adaptive immune system (8). However, there is not much evidence demonstrating the clearance of neoplasms being directly mediated by the complement system. Instead, a tumour-favouring environment arbitrated by the activation of the complement system has been reported (9–12).

**Figure 1:**
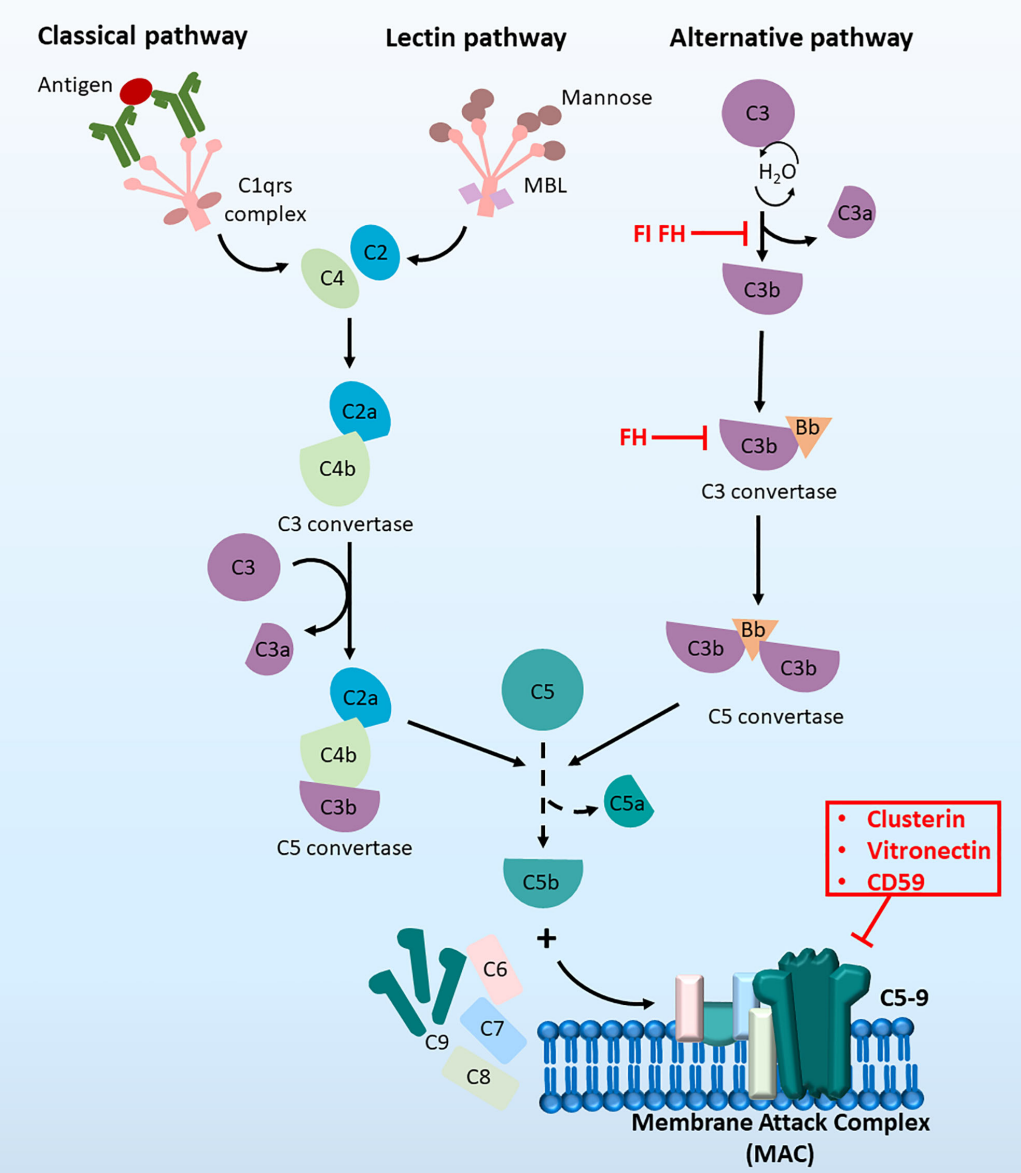
The complement cascade at a glance. The complement cascade can be activated by three pathways, (1) The classical pathway, (2) the lectin pathway and (3) the alternative pathway. Initiation of all three pathways can lead to the formation of C5 convertase to produce C5b, which then recruit C6, C7, C8 and C9 molecules. Together these components can form the terminal membrane attack complex (MAC), which induce cell lysis by inserting pores in cell membranes.

The complement system can be activated by three distinct pathways: the classical pathway, lectin pathway and alternative pathway (13, 14) (**Figure 1**). Both the classical and lectin pathways are activated by the formation of binding complexes (antigen binds to the C1 complex in the classic pathway, and mannose-binding lectin binds to mannose in the lectin pathway), leading to the production of the C3 convertase C4bC2a and ultimately resulting in the assembly of the membrane attach complex (MAC) (15). However, in the alternative pathway, autoactivation occurs by slow hydrolysis of C3. C3 (H_2_O) then binds factor B to form C3 (H2O) Bb, which functions as the C3 convertase in the alternative pathway. The activation of the central component C3 in all three pathways leads to the complete stimulation of the complement cascade (16). Complement regulators are a group of inhibitors of both soluble (15, 17, 18) and membrane-bound forms (19–21). They regulate the complement system by inactivating proteins involved in the cascade, destroying C3 convertase and modulating MAC formation (22). The involvement of the complement system in HCC has been explored but is not thoroughly understood. There is some evidence indicating the therapeutic possibility of complement components as biomarkers or targets for immunotherapies. The involvement of each complement component in the development of HCC and the related therapeutic implications were comprehensively reviewed by Malik and colleagues in 2018 (23). Here, we summarise the recent findings of the complement system in the different hallmarks of HCC as well as its applications in clinical settings.

### Tumourigenesis

Malignancy develops from a complex biological process in which normal cells transform into cancer cells due to the accumulation of genetic and epigenetic alterations resulting in uncontrolled cell proliferation. The local microenvironment also provides a tumour-favouring niche and protective conditions for the growth of cancer cells. The complement system is generally considered a protective mechanism against tumour formation. Complement factor H-related 3 (CFHR3) is a complement regulator that belongs to the human factor H protein family (24). CFHR3 inhibited HCC proliferation and induced apoptosis *via* downregulation of Ki67, Bcl-2 and survivin and upregulation of Bax and active caspase-3 in an *in vitro* model (25). A circulating protein, apoptosis inhibitor of macrophages (AIM), was reported to inhibit steatosis-associated HCC by interfering with the accessibility of regulators of complement activation. By accumulating on the surface of HCC cells and activating complement cascades, AIM eliminated cancer cells by recruiting tumour cell-killing C3. The findings of that study also revealed a novel AIM-based therapeutic strategy for HCC in an animal model (26).

However, more studies revealed a protumourigenic potential of complement in humans. C3a and C5a are derived from the cleavage of complement C3/C5 involved in anaphylatoxic reactions and inflammatory responses *via* direct damage to the cell membrane or by indirect binding to the G protein-coupled receptor C3aR/C5aR on the cell surface. The inhibition of cell proliferation and epithelial-mesenchymal transition in HCC by downregulation of C3aR/C5aR indicated the critical role of complement in tumourigenesis (27). Complement factor H (CFH) regulates alternative pathway activation *via* its ability to bind to self-surface ligands. Laskowski and her research team noticed unexpected spontaneous liver tumour formation in CFH-deficient male mice when they studied complement-related kidney disease (28). CFH deficiency-related HCC may occur due to chronic activation of the alternative pathway, which leads to hepatocellular inflammation and injury and subsequent chronic liver damage and steatosis. Our group also reported that CFH-enriched small extracellular vesicles (sEVs) promoted HCC cell growth, migration, and invasiveness and enhanced liver tumour formation in mice (29). Moreover, our study also revealed the role of CFH-enriched sEVs in inhibiting complement-mediated cytotoxicity, thus facilitating the survival and proliferation of HCC cells. Hepatic stellate cells (HSCs) are one of the most important components in the tumour microenvironment of HCC (30). Active HSCs produce abundant cytokines, including C3 convertase, which plays a critical role in HCC (31). HSCs promoted HCC through C3-mediated suppression of dendritic cell differentiation and enhancement of myeloid-derived suppressor cells. In addition, T-cell apoptosis was exacerbated, and the proliferation of CD4^+^ and CD8^+^ T cells was inhibited under the influence of C3 produced by HSCs (32).

### Metastasis

Liver cancer cells utilise their intrinsic properties to invade adjacent tissues and extravasate the vasculature, metastasize to distant organs and ultimately colonise those organs. The evolved migratory and invasive abilities of liver cancer cells are crucial for the successful liver tumourigenesis and metastasis. Complement participates in the progression of liver cancer and plays a dual role in this process.

All three pathways in the complement cascades converge towards the activation of the major component C3. C3a is generated by C2 cleavage and exhibits a proinflammatory function by binding to C3aR, which is an anaphylatoxin. Using an *in vitro* cell model, the activation of the C3a/C3aR in complement system was revealed to be involved in aristolochic acid I (AAI)-induced cell migration and invasion in HCC (33). The elevated expression of snail may serve as a downstream effect of C3a/C3aR signalling in AAI-induced cell migration and invasion. In addition to C3a, Lee JH’s team revealed that the first component of the classic pathway of complement, C1q, promotes HCC cell motility and invasiveness by directly binding to the collagen receptor discoidin domain receptor 1 (34). Upregulation of complement component C5a is associated with increased expression of TGFβR3 in HCC (35), and it contributes to poor clinical outcomes and promotes tumour progression by activating tumour-promoting macrophages.

A recent clinical study showed that compared with normal liver tissues, HCC samples contained significantly downregulated complement genes: C1R, C6, C7, CFP, and CFHR3 (36). Moreover, the downregulation of the mentioned complement genes was significantly correlated with overall survival, disease‐free survival, and progression‐free survival as well as advanced cancer stages and higher tumour grades in HCC patients. Using bioinformatics analysis of a dataset collected from The Cancer Genome Atlas database, Liu and colleagues revealed that low CFH expression was associated with poor overall survival and relapse-free survival, suggesting that low CFH expression was an independent predictor of poor prognosis in HCC (37). Significantly lower expression of complement component 2 (C2) was found in HCC patients than in healthy controls, and the expression of C2 was associated with TNM stage. Higher C2 expression was significantly associated with better prognosis, and multivariate analysis showed that C2 was also an independent factor for the prognosis of HCC (38). Complement C8 is a main component of the membrane attack complex, and high expression of one of its subunits, C8B, is correlated with better overall survival and recurrence-free survival in HBV-related HCC patients (39), suggesting that the expression of C8B could serve as a good prognostic indicator in this group of HCC patients. The multiple roles of the complement system in cancer progression make it a good candidate for targeted therapies to improve the management of cancer patients. However, there are inconsistent results from different studies, which may be due to the various parameters adopted, and more clinical and basic experiments are required to provide a clearer picture of the role of complement in HCC.

### The complement system and stemness of HCC

Cancer stem cells (CSCs) are the most resilient subset of cells that can undergo self-renewal and differentiation. Compared to their non-CSC counterparts, CSCs show an enhanced capacity for self-renewal, metastasis, drug resistance and immune tolerance (40, 41). Cancer cells display dynamic differentiation states, and the plasticity of CSCs relies on their interaction with various components of the tumour microenvironment (42). Given the involvement of the complement system in tumour microenvironment remodelling, complement proteins might also contribute to the maintenance of stemness in HCC. Seol and colleagues found that complement protein C7 and the complement regulatory protein CFH were upregulated in tumorspheres, a type of CSC surrogate, raised from both primary patient-derived liver tumour cells and liver cancer cell lines (43). Mechanistically, C7 and CFH maintained stemness properties and transactivated the expression of stemness genes by upregulating LSF-1. On the one hand, LSF-1 can transactivate the expression of CFH. On the other hand, osteopontin (OPN), another downstream target of LSF-1, can inhibit the lytic activity of the alternative complement pathway by binding to CFH on the cell surface and therefore prevent tumour cells from immune surveillance, indicating the complex interaction between LSF-1 and CFH (44, 45). CSCs share similar characteristics with normal stem cells, such as self-renewability. Transformation of liver progenitor cells was thought to be one of the origins of liver CSCs (46). One of the components of the C1 complex in the classical pathway, C1q, has been recently reported to support the stemness properties of hepatic progenitor cells. In a conditional β-catenin knockout mouse model, the depletion of β-catenin in hepatocytes created an inflammatory environment with increased secretion of complement C1q from macrophages, which in turn activated the β-catenin pathway of periportal hepatic progenitor cells and led to their expansion and dedifferentiation (47). This finding implicates the role of C1q in supporting the self-renewal ability of stem cells through the β-catenin pathway. C1q was also shown to activate the canonical Wnt signalling pathway through binding to frizzled receptors to promote the impairment of muscle regeneration related to ageing, further supporting the role of C1q in activating the Wnt/β-catenin pathway (48). C3a, the active form of C3, has been found to be elevated in HCV-related HCC patients and proposed to be a novel diagnostic marker for HCV-HCC, although its involvement in CSC regulation of HCC remains unknown (49, 50). The role of C3a in stemness has been implicated in cutaneous squamous cell carcinoma (cSCC). In cSCC cell lines and a xenograft model, C3a was reported to activate the expression of SOX2 and to support stemness by binding to its receptor, C3Ar, and activating the Wnt/β-catenin pathway (51). C5aR, the receptor of C5a, was found to be upregulated in HCC cell lines and tissues, which promoted HCC invasiveness by activating ERK1/2 signalling *in vitro* (52). In human induced pluripotent stem cells (hPSCs), the activation of C5aR1 by C5a stimulated the ERK1/2 signalling pathway and maintained the pluripotency states of OCT-4-positive hPSCs (53). In glioblastoma, C5a secreted from mesenchymal stem-like cells promoted aggressiveness by activating the p38 MAPK/ZEB1 pathway, as demonstrated by cell line models and xenograft models (54). While the role of C1a/C5a and their receptors in CSC maintenance of HCC remains to be explored, the results of these studies implied that anaphylatoxins have a supportive role in regulating stemness pathways and pluripotent genes *via* complement cascade-independent mechanisms by binding to their corresponding receptors. Elucidation of the role of anaphylatoxins in HCC stemness and exploration of complement cascade-related regulatory mechanisms in CSC maintenance would expedite our understanding of the role of the complement system in HCC pathogenesis.

Activation of the complement cascade is controlled by complement regulatory proteins (CRPs). Clusterin, one of the CRPs, has been shown to promote the CSC properties of HCC, including chemoresistance, metastasis and tumourigenesis, by activating the AKT/GSK-3β/β-catenin axis, as demonstrated by *in vitro* and *in vivo* assays (55). Clinically, coexpression of clusterin and β-catenin predicted poor survival. Consistently, by using HCC cell line models, Zhong and colleagues reported that suppression of clusterin sensitized HCC cells to sorafenib treatment by targeting ERK1/2 signalling (56). However, whether clusterin maintains the CSC phenotypes of HCC *via* suppression of complement cascade activation remains to be explored.

### The complement system and the immune suppressive microenvironment of HCC

The complement system is a conventional defence mechanism that links the innate immune response to the adaptive immune response. Activation of complement proteins was thought to be a tumour surveillance mechanism, given the fundamental role of complement proteins against noxious pathogens and the clinical benefit of mAb-based immunotherapy by triggering complement-dependent cytotoxicity towards tumour cells (57, 58). However, studies in recent years have realised that the aberrant activation of the complement system plays an important role in creating an immunosuppressive tumour microenvironment by recruiting immunosuppressive immune cells, inducing immune cell differentiation, upregulating the expression of immune checkpoint molecules and suppressing T-cell toxicity.

Upregulation of C3- and C5-related complement components facilitates the creation of an immune-suppressive microenvironment. The active forms of complement proteins C3 and C5 (C3a and C5a) are potent anaphylatoxins that recruit immune cells, indicating their potential involvement in promoting immune cell infiltration in the tumour microenvironment. A pan cancer multiomics analysis showed that the expression of C3/C5/C3AR1/C5AR1 is associated with the immune evasion signature, indicating the possible immune modulating role of complement proteins in HCC (59). Myeloid-derived suppressor cells (MDSCs) are suppressive immune cells that protect cancer cells from T-cell toxicity. In both an *in vitro* model and an orthotopic HCC transplantation model, C3 from hepatic stellate cells was found to create an immune-suppressive microenvironment by inducing the expansion of MDSCs and promoting the apoptosis of T cells, which facilitated the development of HCC (32). In an orthotopic HCC mouse model, Wang and colleagues reported that PIWIL1-mediated increased secretion of C3 from HCC promoted the infiltration of MDSCs in the tumour microenvironment of HCC *via* the p38/MAPK pathway, which suppressed T-cell proliferation and facilitated HCC development (60). C3a has also been reported to recruit immune suppressive macrophages and neutrophils in other cancers. Tumour-derived C3a was reported to polarize tumour-associated macrophages (TAMs) towards an immunosuppressive M2-like phenotype and to mediate the suppression of T-cell proliferation through the C3a-C3aR axis in a syngeneic mouse model derived from mouse melanoma and in colon and lung cancer cells. Depletion of C3 in tumour cells sensitizes the tumour to anti-PDL1 treatment (61). Tumour-associated neutrophils (TANs) have been known to have protumourigenic effects by mediating immunosuppression to promote metastasis. Hsu et al. reported that C3a promoted the infiltration of immature low-density neutrophils (iLDNs), a protumourigenic neutrophil subtype, in breast cancer-derived liver metastases through the C3a-C3aR axis, as demonstrated in a liver metastasis mouse model created by intrasplenic injection of breast cancer cells (62). These findings imply the potential role of C3a in modulating macrophage and neutrophil function in HCC. While little is known about the immune modulating role of C5/C5a in HCC, the impact of C5/C5a on the immune microenvironment has been evident in other cancers. In colorectal cancer (CRC), C5a/C5aR1 promoted the initiation of CTCs by recruiting MDSCs and impairing CD8^+^ T-cell function (63). C5aR deficiency in mice impaired the liver metastasis of colon cancer by inhibiting the M2 polarisation of TAMs. In contrast, C5a stimulated the M2 polarisation of TAMs through C5aR/NF-κB (64). In clinical prostate cancer tissue, the expression of C5aR was upregulated, which correlated with the expression level of PD-L1. Treatment of prostate cancer cells with C5a stimulated PD-L1 expression (65). C5a and C5aR have been reported to have protumourigenic roles in HCC, indicating their potential involvement in immunomodulation of HCC (52).

In contrast to the possible immune suppressive role of C3 and C5, the expression of some of the complement components might be negatively correlated with the immune suppressive microenvironment. By analysing transcriptomic HCC data from the TCGA database, complement C2 was found to be downregulated in HCC. High expression of C2 was correlated with better HCC survival, with an increased infiltration of CD4^+^ T cells, while a low level of C2 expression was correlated with M0 macrophage infiltration (38). Since reduced infiltration of CD4^+^ cytotoxic T cells was correlated with poor survival of HCC (66) and the potential polarisation of M0 macrophages into protumourigenic M2 macrophages, downregulation of C2 may have an immunosuppressive role in HCC. Mannose-binding lectin (MBL) is an activator of the lectin pathway of the complement system. Using an orthotopic HCC model generated from MBL knockout mice, Li et al. reported that MBL deficiency in mice facilitated HCC tumourigenesis and increased MDSC and Treg infiltration with a reduced percentage of IFN-γ^+^CD8^+^ T cells. Mechanistically, MBL suppressed HCC progression by interacting with HSCs, thus preventing their activation by downregulating the ERK/COX-2/PGE2 pathway (67).

Collectively, dysregulation of the complement system exerts an immune-modulating effect during the progression of HCC. A better understanding of the regulatory mechanism of the complement system on the immune microenvironment of HCC would help to discover novel treatment strategies for HCC patients.

## Conclusion

**Figure 2:**
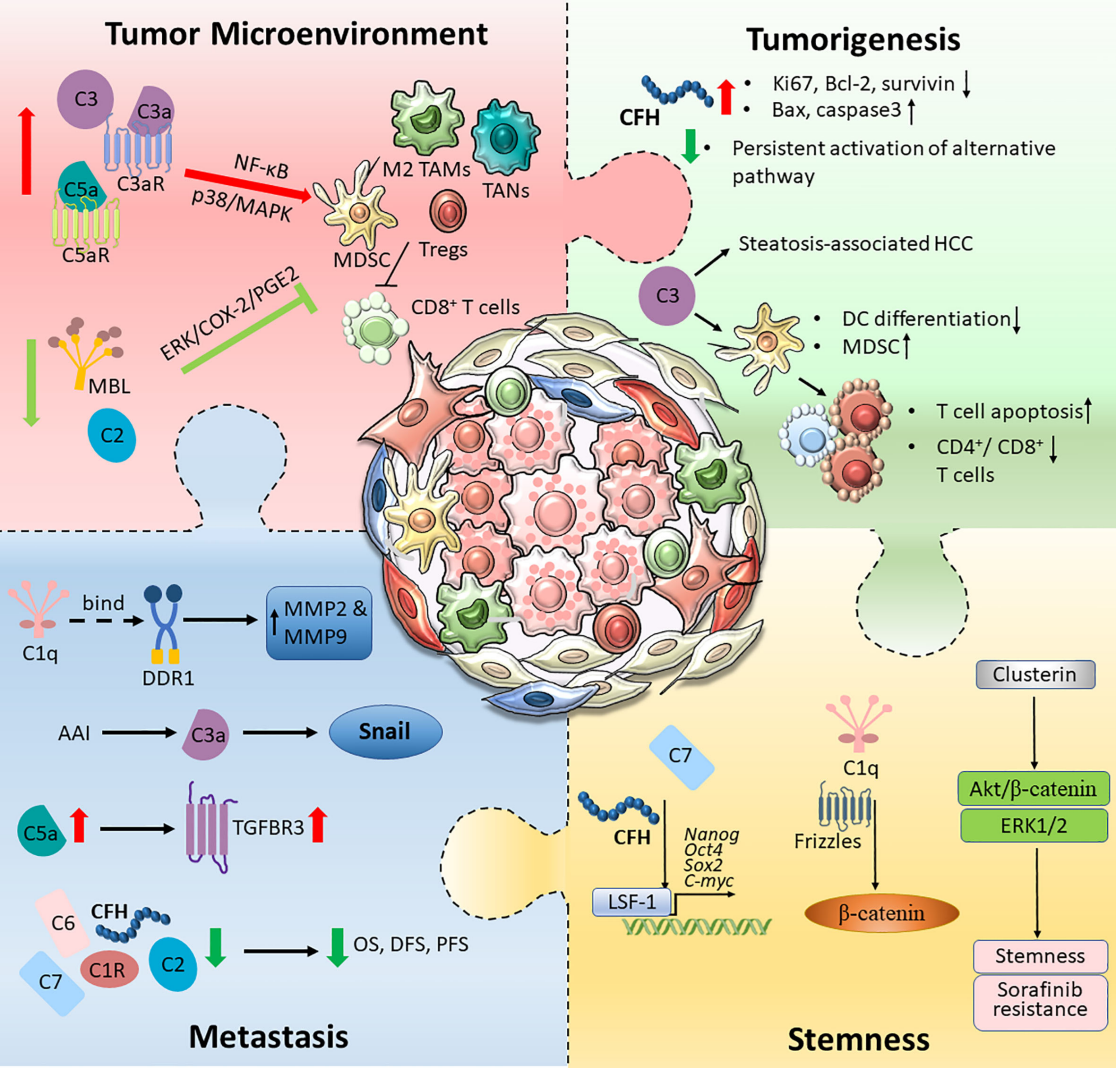
An overview of the complement activation in HCC tumour microenvironment. Tumour cells are capable of hijacking the immune system to cultivate a favourable TME. Different components of the complement pathway were found to be triggered and modified, forming an immunosuppressive environment. The schematic illustration summarised intricate interactions between tumour cells and the complement system, which promotes HCC tumourigenesis, metastatic and stemness.

The aberrant expression of proteins in the complement system plays a complex role in the development of HCC by affecting multiple properties of cancer cells with both protumourigenic and antitumourigenic effects. The expression levels of C1q, C3/C3a, C5/C5a and the regulatory protein clusterin were upregulated in HCC, and this upregulation is responsible for aggressive tumour phenotypes, including tumourigenesis, metastasis, stemness and immune suppression, indicating the potential of these molecules as biomarkers and therapeutic targets for HCC. C1R, C2, C6, C8, MBL, CFP and CFHR were downregulated in HCC and exhibited tumour-suppressive effects; thus, they could serve as prognostic markers for HCC. However, the roles of some complement proteins, including C7 and CFH, are controversial. Some studies have reported their downregulation and antitumourigenic role in HCC, while others have illustrated their roles in supporting the invasiveness and stemness of cancer cells; thus, they have differential roles in the progression of HCC (**Figure 2**). While our knowledge of the detailed regulatory mechanisms of complement proteins in the pathogenesis of HCC is still limited, the reported findings implied multifaceted roles of the complement system in HCC. A deeper understanding of the mechanistic interaction between the complement system and HCC would foster the development of a novel therapeutic strategy for HCC targeting complement, either as a single treatment or in combination with traditional chemotherapies, targeted therapies or immunotherapies.

## Author contributions

ZJX contributed to the conception and design of the article, drafted the article and interpreting the relevant literature. CLSY drafted the figures and interpreting the relevant literature. JWPY revised the article critically for important intellectual content. XWM contributed to the conception and design of the article, drafted the article and interpreting the relevant literature. All authors contributed to the article and approved the submitted version.

## Funding

National Natural Science Fund under National Natural Science Foundation of China [Project no. 81872340]. Research Start-up Fund of the Seventh Affiliated Hospital, Sun Yat-sen University [Project no. ZSQYBRJH0023].

## Conflict of interest

The authors declare that the research was conducted in the absence of any commercial or financial relationships that could be construed as a potential conflict of interest.

## Publisher’s note

All claims expressed in this article are solely those of the authors and do not necessarily represent those of their affiliated organizations, or those of the publisher, the editors and the reviewers. Any product that may be evaluated in this article, or claim that may be made by its manufacturer, is not guaranteed or endorsed by the publisher.
